# Should GPS data be normalized for performance and fatigue monitoring in soccer? A theoretical-practical discussion on high-speed running

**DOI:** 10.3389/fspor.2025.1603767

**Published:** 2025-06-25

**Authors:** Ricardo Pimenta, Hugo Antunes, João Ribeiro, Fábio Yuzo Nakamura

**Affiliations:** ^1^ Research Center in Sports Sciences, Health Sciences and Human Development (CIDESD), University of Maia, Maia, Portugal; ^2^ Research Center of the Polytechnic Institute of Maia (N2i), Maia Polytechnic Institute (IPMAIA),Castêlo da Maia, Maia, Portugal; ^3^ Department of Rehabilitation and Performance Optimization (DROP), Futebol Clube Famalicão - Futebol SAD, Famalicão, Portugal; ^4^ FSI Lab, Football Science Institute, Granada, Spain; ^5^ Department of Performance Optimization (GOD), Sporting Clube de Braga SAD, Braga, Portugal

**Keywords:** high-speed running, soccer, load monitoring, GPS, speed thresholds

## Abstract

High-speed running (HSR) is one of the performance metrics of interest, as the volume of HSR during matches has been increasing over the last decade, which suggests that weekly training loads should be adjusted to align with this trend, enabling players to cope with match demands. However, the use of HSR thresholds lacks a solid rationale for their application and fails to account for individual player capacities, likely not reflecting their actual HSR efforts. As such, this theoretical-discussion provides important implications for training prescription, aiming to optimize performance and minimize fatigue. It emphasizes the significant differences in the conceptualization of HSR and highlight**s** the advantages of adopting a normalized approach that reflects the physiological, mechanical and neuromuscular aspects related to HSR, as well as the intermittent profile of football matches. Practical HSR threshold definitions tailored to the capacities of each athlete are proposed, enabling a more evidence-based approach for the interpretation of training loads and game/player profiling. More specifically, within our proposal, HSR can be subdivided into two types: (1) HSR-1, characterized by an entry threshold based on a normalized critical speed, and (2) HSR-2, defined by an entry threshold corresponding to 75% of the athlete's maximum speed.

## Introduction

The Global Positioning System (GPS) was first used for athlete tracking in 1997 (1). Since then, its utilization has spread across various sports, including soccer, enabling real-time analysis of players’ on-field activity profiles during training and competition (2). The activity profile includes various metrics related to various aspects of the athlete's physical performance, such as running performance. It has been observed that match intensity in male soccer, in terms of high-speed running (HSR) metrics, has increased over the last decade (3, 4). Such increases suggest that players probably require training adjustments to align with the evolving physical demands of soccer matches (5). Indeed, previous studies has focused on examining the effects of various game-based formats (e.g., small-sided and large-sided games) on both external and internal load metrics, owing to the widespread implementation of such formats in training sessions across the microcycle and their relevance to soccer-specific demands (6–8). However, adequate adjustments warrant appropriate data analysis and interpretation, which are fundamental steps preceding the operationalization of the training stimulus. In this context, a problem arises when considering what constitutes realistic efforts in terms of the actual workloads experienced by the athletes. The fixed thresholds for HSR are set between 14.4 km/h and 21.1 km/h for males, and between 12.2 km/h and 15.6 km/h for females, with the most common thresholds being 19.8–25.2 km/h for males and 12.5–22.5 km/h for females which results in approximately 1,000 m and 760 m for professional female and male players, respectively (9). However, the rationale behind the conceptualization of these thresholds remains unknown. The use of such thresholds may hinder coaches from providing tailored training stimuli to players, thereby failing to achieve the desired adaptations and potentially increasing the risk of injuries (10, 11). Considering the principle of individualization, the creation of relative thresholds would address this issue. This review substantiates the importance of considering the normalization of HSR data for an adequate load management in male soccer players.

### The problematic applicability

**Figure 1:**
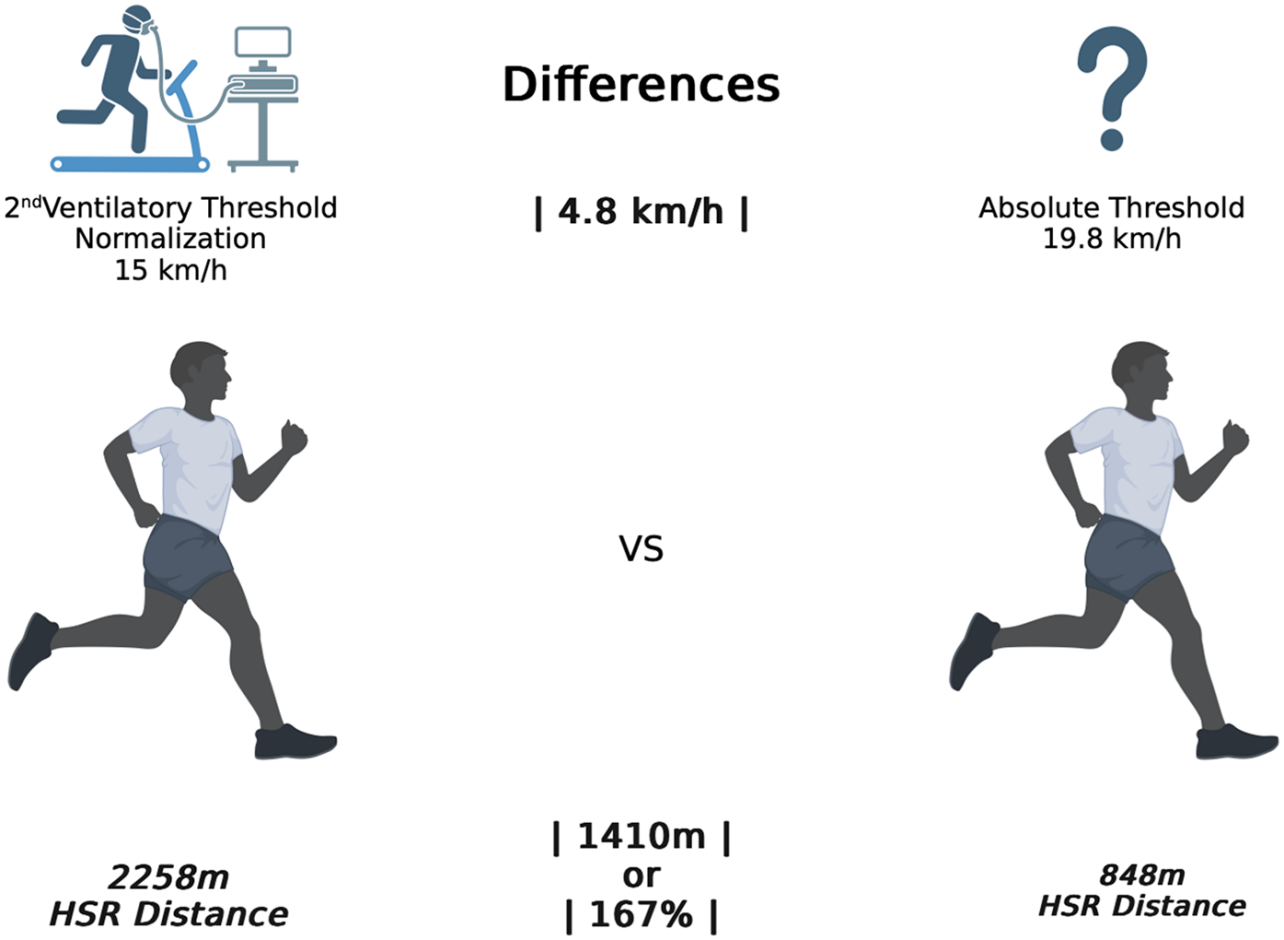
Comparison between HSR distances using the second ventilatory threshold (15 km/h) and the absolute threshold of 19.8 km/h in soccer male athletes.

Erroneous interpretations of the external load imposed on the athletes might lead to inadequate training exposure, resulting in insufficient or excessive stimulation for optimal training adaptations or overtraining (10, 11). In theory, the use of normalized speed thresholds should contribute to better approximating the workload applied to individual athletes in relation to the intended physiological stimulus. With training individualization being one of the fundamental principles of exercise training, it is expected that normalization of the threshold values according to the individual's maximum or relative capacities would be an approach that more closely resembles the real physiological impact experienced by the athletes. Previous studies linked the second ventilatory threshold (VT2) (point at which occurs a shift in the ventilation strategy with an exponential increase in ventilations per minute relative to oxygen uptake) to the corresponding speed attained at that specific moment, using it as the reference value for HSR entry (12, 13). This speed was approximately 15 km/h, which was 4.8 km/h lower than the 19.8 km/h absolute threshold. In those studies, HSR distance > 15 km/h (normalized value) was 2,258 m, compared to 845 m covered using the 19.8 km/h absolute threshold value, representing a 167% increase in HSR distance when using a normalized approach. This example is illustrated in Figure 1.

In addition, utilizing the VT2 as reference for the lower HSR threshold signifies that a speed amplitude of 10.2 km/h from 15 to 25.2 km/h, within this HSR range will reflect substantially different kinematic patterns experienced by the athletes (14, 15).

A proper definition of running intensity would encompass the physiological, mechanical, and neuromuscular domains of physical exertion. As running speed increases, the changes in the running pattern (16), VO_2_ kinetics (17) and neuromuscular activation of specific muscle groups (18) become more noticeable. Considering these responses collectively provides a more nuanced perspective on how to establish an HSR threshold that accurately reflects the effort experienced by the athlete.

Substantial differences arise between the application of absolute and normalized threshold values in defining HSR entry probably leading to misinterpretations of the athletes’ exertion further compromise the accuracy of training prescriptions and the validity of game and training analyses (13, 19). Moreover, there remains a lack of conclusive evidence supporting the superiority of normalized over absolute thresholds for HSR efforts in soccer. Further investigations are required to identify the most appropriate method for assessing and establishing normalized thresholds.

### Physiological domain

The player's aerobic capacity is frequently evaluated as it is recognized as a key physical attribute for success in team sports (20). Various protocols have been used to assess this physical capacity in team sports; however, they may capture subtle yet significantly distinct physical characteristics (21), which can prevent coaches from making direct comparisons between results obtained from different protocols, even when used for the same purpose.

Researchers have considered the HSR definition as the speed corresponding to maximal oxygen consumption (VO_2_max) (22), reporting running speeds of approximately 16.2–16.5 km/h at VO_2_max in professional soccer players (23). A higher VO_2_max has been related to the player's capacity to cope with match demands (24) and a faster recovery between high-intensity actions (25). Furthermore, VO_2_max may be similar between players with distinct running performance capacities (26). To differentiate athletes with similar VO_2_max values, physiological cut-points may be utilized, serving as running-intensity thresholds and representing key speed markers attained by the athlete, such as maximal aerobic speed (minimum running speed at which maximum oxygen uptake occurs) or the maximum lactate steady state (highest exercise intensity where a balance is observed between the rate of production and removal of blood lactate).

Typically, three physiological domains are used to characterize training intensities from moderate, heavy and severe (27). Nevertheless, the assessment of physiological cut-points commonly encompasses logistical and methodological limitations. Commonly, these physiological cut-points are assessed during a laboratory treadmill test (28), which are considered non-ecologically valid since soccer is profiled as an intermittent sport (20). Moreover, running kinematics and kinetics are affected by increasing speed, which has repercussions at the neuromuscular and mechanical levels, and their relationship with the physiological cut-points remains unclear. Tests considered more ecological, such as the 30-15 Intermittent Fitness Test (30-15_IFT_), have been conducted to assess aerobic fitness and intermittent exercise capacity related to gameplay (29) incorporating speed and change of direction abilities, along with lower-limb power and inter-effort recovery (29). Although the final speed of the 30-15_IFT_ appears more appropriate as a threshold for HSR due to its specificity and its closer alignment with the locomotor profile observed in soccer, it must be acknowledged that there is currently no established method to validate it for this purpose. Since locomotion at varying intensities in soccer does not occur over standardized distances and trajectories, it is not feasible to establish a field test that accurately determines the onset of high-intensity running. Consequently, selecting a test to define the cut-off point will inevitably involve an inherent degree of arbitrariness. Partially solving these issues, validation of soccer match GPS-derived critical speed (CS) estimates between 13.7 and 14.4 km/h have been reported (30) recurring to a mathematical time-based modeling of competition games and CS field tests for posterior correlation analysis, withdrawing the necessity of conduction specific and time consuming tests. This may also apply for the assessment of other running performance metrics such as maximal speed (31). Therefore, CS could be a plausible cut-point of a physiological-based lower HSR threshold since it is intimately associated with each player's running capacity.

### Kinematic and mechanical domain

The progressive increase in running speed is associated with an evolving running pattern marked by significant kinematic differences (14). It has been suggested that HSR may be more accurately described as velocities near 75% of the maximum speed, as this better reflects relative speeds associated with individual striding patterns (16). Given that HSR (considering speeds between 19.8 km/h and 25.1 km/h) accounts for approximately 7%–11% of the total distance covered during a soccer match (3), the majority of the distance covered is associated with lower-intensity movement patterns, such as running, jogging, or walking. However, while 75% of maximum speed represents a striding pattern for the sample observed in that study, it is possible that this finding may not be generalizable to professional soccer players. At a neuromuscular and mechanical level, this could have significant implications. Indeed, previous studies report neuromuscular and mechanical repercussions of increasing running at the hamstrings muscle group. The peak musculotendinous stretch of the hamstrings was observed to occur at 80% of the peak speed (32), while further increases in speed were related to a higher negative work of the hamstrings (33, 34) and a peak neuromuscular activation of the hamstrings at 90% peak speed (35). However, it is important to note that these observations are specific to the hamstrings and may differ for other muscle groups, such as quadriceps and calf musculature (15). Altogether, current data regarding neuromuscular, mechanical and kinematic parameters of running speed, abet the idea that 75% peak speed might be an adequate starting point to observe a HSR mechanical-oriented stimulus with an upper ceiling of 90% peak speed, from which the players will adopt maximal sprinting kinematics (16). Indeed, a recent study developed the rationale regarding the normalization of maximal sprinting speed >90%, when compared to absolute thresholds (36). Furthermore, 75% and 90% peak speed represent mean values of a given sample, as so, it is expected to prognosticate variations according to the peak speed values of each athlete. Even so, since they were obtained through an individualization procedure, these peak speed mean values probably provide a better approximation to the real HSR efforts compared to the commonly used arbitrary thresholds, reinforcing the need to assess HSR kinematics of each individual.

### Practical application—refining speed thresholds in soccer

Different studies have used different physiological approaches to relate speed thresholds (12, 37). Nevertheless, the protocols applied fail to adequately represent the intermittent and repeated acceleration associated with soccer games (38), and therefore, can be deemed as inaccurate tests to generate tailored speed thresholds. The use of speed thresholds that do not adequately fit the real physiological and mechanical effort exerted by athletes leads coaches and sport scientists to erroneous interpretations, which will likely result in inappropriate training monitoring, potentially impacting fatigue management of the squad and increasing the injury risk, not only in healthy players but also in those undergoing rehabilitation processes (10, 11).

Due to the aforementioned variables, justifying the generation of a proper normalized HSR threshold is a very complex task. Still, we do consider that some variables could give us insights into, or at least, define the boundaries of HSR intensities. The CS corresponds to the highest running speed value that an athlete can sustain without a significant fatigue accumulation (39) closely related to the second lactate threshold or to the severe intensity exercise domain, since running beyond the CS will result in a rapid accumulation of lactate, making it impossible for the athlete to maintain that running speed for long. Regardless of the running mechanics associated with the CS, we can be confident that above this threshold, the athletes will develop significant levels of fatigue, as has been observed elsewhere (40).

**Figure 2:**
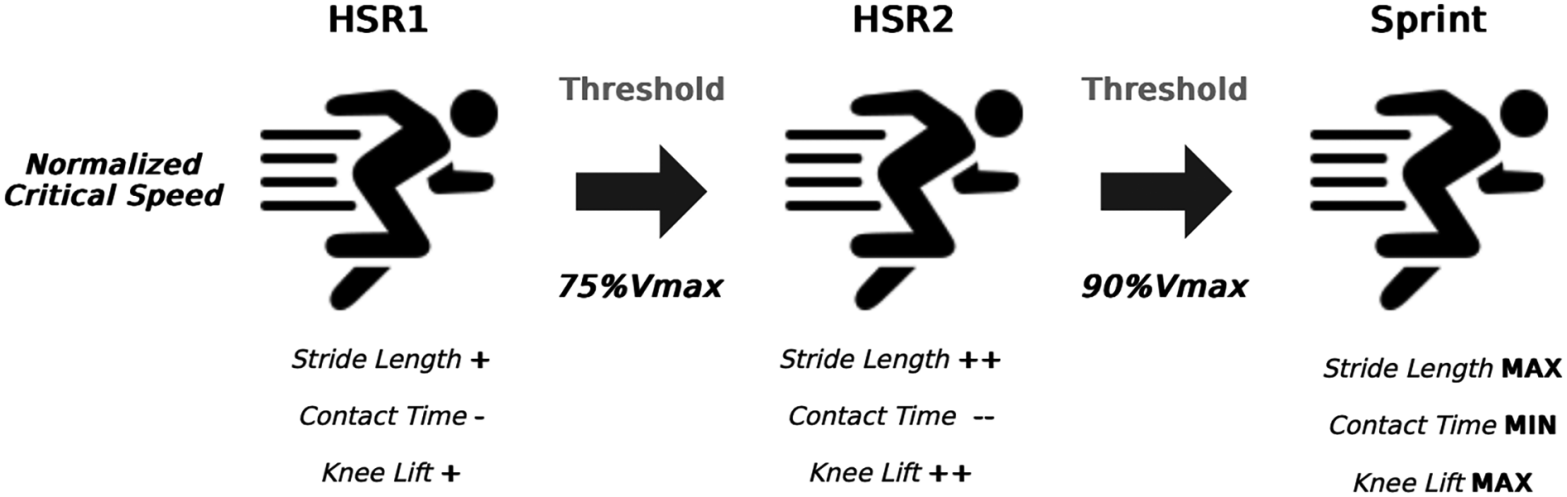
Kinematic differences between different high-speed running types (1 and 2) and sprint and their respective thresholds. HSR1 (metabolic) intensity initiates above the normalized critical velocity, HSR2 (mechanical) intensity initiates above the 75% maximum velocity and sprint intensity initiates above 90% maximum velocity. (+) and (-) signs, represent an arbitrary emphasis regarding the quantity of the observed variable.

As HSR speed continues to increase above the CS, not only will fatigue develop more rapidly, but the running patterns will progressively approximate into a sprinting pattern, from running to striding and near maximal sprinting. Since all patterns comprise speeds above CS, a kinematic-based approach to define the upper limit could eventually be considered as an eventual solution. The altered running kinematics approximating the sprint kinematics will be associated with neuromuscular activation and mechanical strains experienced by the muscles that are significantly superior compared to running speeds closer to the lower HSR threshold. Since maximum neuromuscular hamstrings activation occurs at 90% maximum sprint speed (35), this could be applied as the maximal HSR threshold. Unfortunately, there is not sufficient evidence to justify an intermediate threshold between the upper and lower limits of HSR intensities, besides the observations of 75% of maximum speed representing a striding pattern in a group of amateur field-sports athletes (16). This rationale of a two-zone HSR intensity, separating a more cardiometabolic type (intensities closer to the CS) to 75% of maximal speed and a more mechanical type (intensities between 75% of maximal speed and 90% of maximum sprint speed), paves the way for an HSR spectrum construct that will aid in the analysis of the running load profile of the soccer players (Figure 2). Considering the actual thresholds, if a player covers a certain number of meters in HSR, using the arbitrary threshold of >19.8 km/h, we will likely miss a great cardiometabolic component that is already present at lower running speeds, since these speeds already elicit significant cardiorespiratory stress (13). On the superior portion of the spectrum, some athletes may actually present an individual level of HSR intensity corresponding to speeds greater than the commonly used arbitrary thresholds (16), resulting in an overestimation of the external workload, and thereby overlooking the actual training load, which could have significant consequences for performance and fatigue monitoring. Further research is needed to understand what variables might help identify the transition between lower HSR intensities and higher HSR intensities. Since both intensities are situated above the CS, variables related to HSR kinematics could provide valuable insights into this complex phenomenon, given that running kinematics are associated with neuromuscular and mechanical demands experienced by muscle groups such as the hamstrings.

## Discussion

Male soccer players of elite status have been observed to cover a total mean distance between 10 and 12 km (41). The majority of the distance is covered below the anaerobic threshold in elite soccer players, with an accepted corresponding running speed of 14.4 km/h (42) which is substantially inferior compared to the absolute threshold (19.8 km/h) commonly characterizing HSR (9). Thus, whenever players cover HSR distances, whether normalized or not, a greater rate of fatigue is expected to develop compared to lower-intensity running speeds especially for slower players whose HSR thresholds are relatively closer to their maximum speed capacity. As running speeds come closer to the upper limit of HSR intensity, a greater effect of fatigue is observed, due to an increased neuromuscular (18) and mechanical effort in muscles of the lower limbs. As running speed increases, eccentric contractions at higher velocities become more frequent (43), particularly in the hamstrings, leading to increased muscle damage (18) and an elevated neuromuscular demand, which likely contributes to reduced neuromuscular performance. As previously reported in the literature, load management might be crucial for performance optimization and/or injury prevention (44). However, the differences between using normalized or absolute threshold are likely to result in disparate load management metrics, ultimately leading to different interpretations of their practical applications. It should be noted that 10% of the total distance covered per match is performed at high-intensity (45) with significant match-to-match variations and player (46). Values reported regarding HSR variables between studies including teams from the same or different competitive leagues should not be generalized or applied in different contexts (47) since tactics, predominant style of play, and players’ quality (in terms of technical-tactical skills) may be significantly distinct (48). For instance, considering a player with a maximum speed capacity of 36 km/h, the absolute HSR threshold of 19.8 km/h represents 55% of their maximum capacity, while for an athlete with a maximum speed capacity of 30 km/h, the same threshold represents 66% of their maximum capacity. Therefore, this suggests that game profiling should consider reporting HSR distances according to their proximity to the lower (CS to75% maximum speed) and upper limits (75%–90%maximum speed) of normalized HSR intensities, as they will reflect different mechanical and neuromuscular impacts.

Moreover, ideal training adaptations require the repeated application of appropriate training stimulus which is compliant with fundamental training principles such as individualization and progressive overload. In order to consummate such principles, coaches need to identify the athlete's maximal capacities and subsequently prescribe training intensities and volumes according to those maximal references. However, external load monitoring recurring to arbitrary speed thresholds precludes coaches not only from properly interpreting external load produced by the athletes but also to prescribe training loads in an individualized manner. In other words, coaches may be erroneously applying training intensities for half of the squad, likely increasing the injury risk occurrence while minimizing the promotion of positive training adaptations. Indeed, appropriate running intensity exposure as is the case for intensities superior to 90% of maximum speed capacity (49), has been reported to enhance sprint performance and simultaneously provide a prophylactic effect for soft-tissue injury (50).

Normalized HSR values for performance and fatigue monitoring could be an advantage for prescribing training loads for each athlete, but it does not facilitate the comparison between athletes. The comparison between athletes requires the use of absolute HSR thresholds to benchmark players’ performance in game or training sessions, providing a collective measure for the team or specific positions, which allows for ranking the athletes. Even if each player's individual capacities are assessed accurately, they may still fall well below the average absolute capacity expected for their field position or team standards. Such inter-athlete comparisons must rely on an HSR threshold value that aligns as closely with the average (albeit unknown) mean of HSR speeds, so as to capture the majority of HSR efforts experienced by players. This approach requires further investigation.

This perspective discussion article substantiates the importance of considering the normalization of HSR data for an adequate load management in soccer players. A two-dimensional conceptualization of HSR is proposed, based on the physiological, neuromuscular, mechanical and kinematic parameters, (1) a metabolic HSR, composed of running speeds between critical velocity threshold and 75% of maximal speed; (2) a mechanical HSR initiating at 75% peak speed with an upper threshold of 90% peak speed, with expected variation according to the peak speed of each player. In practical terms, the current proposal also enables strength and conditioning coaches for a better understanding of the real HSR consequences on fatigue development will contribute to minimizing fatigue and enhancing performance, concomitantly having a possible beneficial impact over the injury risk.

## Data Availability

The original contributions presented in the study are included in the article/Supplementary Material, further inquiries can be directed to the corresponding author.
